# Non-coding RNAs in lung cancer

**DOI:** 10.18632/oncoscience.98

**Published:** 2014-11-15

**Authors:** Biagio Ricciuti, Carmen Mecca, Lucio Crinò, Sara Baglivo, Matteo Cenci, Giulio Metro

**Affiliations:** ^1^ Medical Oncology, Santa Maria della Misericordia Hospital, Azienda Ospedaliera di Perugia, Perugia, Italy; ^2^ University of Perugia, Perugia, Italy

**Keywords:** non-coding RNAs, lung cancer, targeted therapy, biomarkers

## Abstract

The discovery that protein-coding genes represent less than 2% of all human genome, and the evidence that more than 90% of it is actively transcribed, changed the classical point of view of the central dogma of molecular biology, which was always based on the assumption that RNA functions mainly as an intermediate bridge between DNA sequences and protein synthesis machinery. Accumulating data indicates that non-coding RNAs are involved in different physiological processes, providing for the maintenance of cellular homeostasis. They are important regulators of gene expression, cellular differentiation, proliferation, migration, apoptosis, and stem cell maintenance. Alterations and disruptions of their expression or activity have increasingly been associated with pathological changes of cancer cells, this evidence and the prospect of using these molecules as diagnostic markers and therapeutic targets, make currently non-coding RNAs among the most relevant molecules in cancer research. In this paper we will provide an overview of non-coding RNA function and disruption in lung cancer biology, also focusing on their potential as diagnostic, prognostic and predictive biomarkers.

## INTRODUCTION

Non-coding RNAs (ncRNAs) represent a novel class of RNA molecules fulfilling many basic regulatory functions in Eukaryotes, and whose dysregulation has been reported in a wide spectrum of human diseases, including cancer.

The discovery that the human genome encodes only ~20,000 protein-coding genes, representing less than 2% of the total genome sequence, and the demonstration that at least 90% of the genome is actively transcribed, suggest that most of the transcriptome is constituted by non-coding RNA [1-4]. These findings have been possible because of the development of tiling resolution genomic microarrays and whole genome and transcriptome sequencing technologies (ENCODE project) [4].

To date, non-coding RNAs primarily include: small non coding RNAs, such as transfer RNAs (tRNAs), ribosomal RNAs (rRNAs), small nucleolar RNAs (snoRNAs), microRNAs (miRNAs), small interfering RNAs (siRNA), PIWI-interacting RNAs (piRNA), antisense RNAs, promoter-associated RNAs (PARs) and different types of long non coding RNAs (Table 1).

**Table 1:** Main classes of human non-coding RNA

**1. Small non-coding RNAs (< 200 nt.)**	
Transfer RNAs (tRNA)	*Short-chain RNA molecules involved in aminoacids transfer for protein synthesis* [7].
Ribosomal 5S and 5.8S RNA (rRNA)	*RNA component of ribosomal subunit involved in protein translation* [8-9]
MicroRNAs (miRNA)	*Class of small non-coding RNAs that targets protein-coding mRNAs at the post-transcriptional level (18-22 nt.)* [10-11].
Small interfering RNAs (siRNA)	*Post-transcriptional gene silencing RNA-mechanism that involves the degradation of messenger RNA in a highly sequence-specific manner (21-22 nt.)* [12]
PIWI-interacting RNAs (piRNA)	*Small endogenous non-coding RNAs that form the piRNA-induced silencing complex (piRISC) in germline development, involved in the regulation of transposons stability and stem-like epigenetic state of cancer cells (26-31 nt.)* [13-14]
Small nuceolar RNAs (snoRNA)	*RNA molecules involved in modification and processing of other RNAs, especially rRNAs, tRNAs and snRNAs mainly by site-specific methylation and pseudouridylation* [15-17]
MicroRNA-offset RNAs (moRNA)	*RNA derived from pre-microRNA with expression levels unrelated to those of the associated microRNAs (20 nt.)* [18]
Promoter-associated short RNAs (PASRs)	*Less than 200 nt. trascripts that may regulate gene expression through the interaction with genes promoter sites* [19]
Small nuclear RNAs	*Non-coding RNA molecules involved in pre-mRNA splicing, and regulation of transcription factors* [20]
Transcription initiation RNAs (tiRNAs)	*18 nucleotides small RNA conserved from insects to mammals involved involved in transcription regulation* [25-26]
**2. Long non-coding RNAs (>200 nt.)**	
Long non-coding RNAs (lncRNA)	*Polyadenylated RNAs greater than 200 nucleotides in length that regulate gene expression through epigenetic regulation, splicing, imprinting, transcriptional regulation and intracellular transport (>200 nt.)* [21]
Antisense RNAs	*Single stranded RNA complementary to transcribed mRNAs, involved in binding and blocking the translation process of the mRNA target* [22]
Promoter associated longRNAs (PARs)	*Longer than 200 nt. trascripts that may regulate gene expression through the interaction with genes promoter sites* [19]
Transcribed ultraconserved regions (T-UCR)	*Highly conserved genomic elements involved in the maintenance of splicing factors expression levels and gene expression regulation* [23]
Pseudogenes	*Non-functional sequences of DNA originally derived from functional genes but with mutations or premature stop codons that avoid their expression. Involved in regulation of gene expression and recombination* [24]
Telomere-associated ncRNAs (TERRAs)	*Negative regulators of telomere length through inhibition of telomerase and involved in telomere associated diseases including many cancers* [27]

It is clear today that these kinds of molecules operate not only as gene expression and splicing regulators, but also as epigenetic controllers and guides for cromatine modifying complexes [1, 5].

Within this class, miRNA, siRNA, piRNAS, and lncRNAs have been recently studied, and many of their functions are well defined; considering the different activities they perform, it is not surprising that their deregulation may be involved in various human diseases including different types of cancer such as lung, breast, colon, liver and prostate [4].

Thus it seems extremely important to consider all the possible applications of these ncRNAs in oncology, as markers of disease and possible therapeutic targets.

Lung cancer is the leading cause of cancer-related deaths worldwide in both men and women, representing 25% of all cancer related deaths. Among lung cancers, 80% are classified as non-small cell lung cancer (NSCLC) and 20% as small cell lung cancer (SCLC) [6]. Although advances in diagnostic techniques and treatments have resulted in an increased survival rate and knowledge of lung cancer biology has also improved, the prognosis remains poor with a 5-year worldwide survival rate of less than 15% [6]. Current research focuses especially on genomic and epigenomic alterations of protein-coding genes that may be predictive of greater or lesser response to anticancer treatments. Without doubt, the discovery of specific genetic alterations such as EGFR mutations, ALK and ROS1 rearrangements, has enormously improved the outcome of patients harboring this specific kind of modification, mainly because of the development of biological target therapies. In this context, the evidence that specific genetic mutations are related to distinct pathological and clinical features has paved the way to the era of personalized therapy [28-30].

If on one hand this has represented a significant change in the management of these patients, on the other hand the recent advances in our knowledge of non-small cell lung cancer pathobiology and genetic assessment requires us to consider other aspects as well, in particular those concerning the finest mechanisms of gene expression and regulation. Moreover, if we consider that most of the efforts made to date in lung cancer research focused on 2% of the entire genome we can easily understand how much more efforts are required and how important is to investigate the non-coding genome universe.

### MicroRNAs and lung cancer

MicroRNA are a class of 18–25 nucleotide long, evolutionarily conserved, endogenous, single stranded small non-coding RNAs, that target protein-coding mRNAs at the post-transcriptional level, binding to the 3′-untranslated regions (3′ UTRs) of mRNAs, which results in target mRNA degradation or translational inhibition [10, 31].

Actually, it is believed that miRNA genes account for only about 1–2% of the human genome, nevertheless they control the activity of approximately 50% of all protein-coding genes [31-33]. In this paragraph we will explore the advancement in human miRNA biology, function and its involvement in lung cancer.

### MircoRNA biogenesis and function

In recent years, much evidence has led to a classification of miRNAs into three main types: intergenic miRNA, coding-intronic miRNA and coding-exonic miRNA [34].

Intergenic miRNAs are transcribed by RNA polymerase II or III and produce an intermediate primary transcript (pri-miRNA); only pri-miRNAs with an appropriate stem length, a flexible terminal loop ≥ 10 bp and the capability of producing 5′ and 3′ single-stranded RNA overhangs can be converted into mature and functional miRNA [35-37]. Then, pri-miRNAs undergo a nuclear cleavage by a multiprotein complex termed microprocessor which includes the RNase III endonuclease Drosha and the double-stranded RNA-binding protein DiGeorge syndrome critical region gene 8 (DGCR8), also named Pasha in invertebrates [37-38]. In particular, data suggests that DGCR8 recognizes the pri-miRNA through the ssRNA-dsRNA junction at the hairpin base and directs Drosha to a specific cleavage site; after this process, RNase III endonuclease Drosha releases a 60- 70 bp miRNA precursor called pre-miRNA [37, 39-40].

Otherwise, miRNAs derived from the intronic regions of protein coding genes are trascribed by RNA polymerase II, the miRNA sequence is excised from the pre-mRNA by spliceosomal complex or the microprocessor and liberate a mirtron or a pre-miRNA. These lariats are debranched and refolded into the typical stem-loop structure of pre-miRNAs, and then enter the classical pathway. Alternatively, a primary miRNA (pri-miRNA) is released and undergoes microprocessor cleavage to generate pre-miRNA [37, 41].

Subsequently pre-miRNAs are transported to cytoplasm by the RAN GTP-dependent transporter exportin 5; in the cytoplasm, pre-miRNAs are first cleaved by RNase III Dicer into a 22 nucleotide long miRNA duplex, and immediately separated by helicase into a passenger strand, which undergoes a degradation process, and a guide strand. More specifically, the guide strand, which results in 18–25 nucleotide long mature miRNA duplex, becomes part of the miRNA-induced silencing complex (miRISC) that also includes the human immunodeficiency virus transactivating response RNA binding protein (TRBP) and the Protein Kinase R-activating protein (PACT) [37, 42-44].

**Figure 1:**
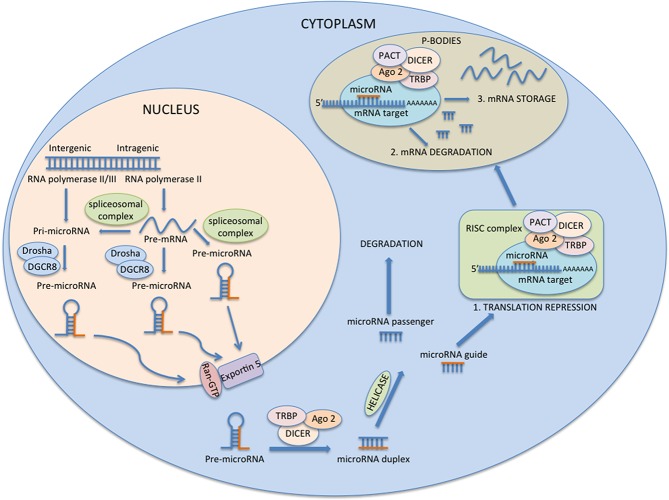
MicroRNAs biogenesis and function miRNAs are transcribed by RNA polymerase II/III and produce intermediate primary transcripts termed pri-miRNAs, which subsequently undergo a nuclear cleavage by a multiprotein complex (Drosha/DGCR8) leading to the genesis of pre-miRNAs. Pre-miRNAs are transported to cytoplasm by the RAN GTP-dependent transporter exportin 5 and are further processed by the enzyme Dicer, resulting into a 22 nucleotide long miRNA duplex formed by a passenger strand and a guide strand. Only the guide strand, which results in 18–25 nucleotide long mature miRNA duplex, becomes part of the miRNA-induced silencing complex (miRISC) and mediates gene silencing by interfering with translational process or inducing mRNA degradation and storage into the P-bodies.

Also worth mentioning is the Dicer-independent pre-miRNA processing model, based on Argonaute 2 protein (Ago2) and proposed by Cifuentes [45]. Ago2 possesses both a Piwi and PAZ domain with endonuclease activity that cleaves the passenger strand of the precursor 10 nucleotides upstream the guide strand 5′; this cleavage lets the nucleotides near the cleavage sites, that are no longer protected by Ago2 binding, to subsequently undergo polyuridylation and nuclease-mediated trimming to achieve mature miRNA [37, 45]. Ago2 is also involved in a different Ago2-dependent pre-miRNA processing path based on the aggregation of Dicer, TRBP and Ago2 in a multimeric complex. Ago2 cleaves a single-strand of the pre-miRNA generating a nicked hairpin structure denominated “Ago2-cleaved precursor miRNA.” This precursor works as a substrate for consecutive Dicer cleavage to generate the double stranded miRNA duplex [45-48]. Once activated, the RISC complex binds the mRNA target via a base pairing mechanism between the miRNA guide and the 3′UTR of the target [49]. Should they be extensively base-paired, target mRNA is cleaved by Ago2 and subsequently deadenylated by a protein complex which includes Pop2, Ccr4 and Not1. At this point mRNA can be degraded from 3′ to 5′ by the exosome complex or from 5′ to 3′ by the Xrn1p exonucleases after undergoing a decapping process by Dcp enzymes. The mechanism just described is known as Slicer-Dependent Silencing [50-51]. But base-pairing between the miRNA guide and mRNA target is limited, and gives rise to a lump in the RNA duplex which does not allow Ago2 mRNA cleavage and results in the Slicer-independent silencing path which is directly miRNA mediated and consists of mRNA accelerating deadenylation and decapping processes, translation repression, spatial separation of translation components and mRNA sequestration into cytoplasmic foci known as P-bodies [52-54]. In fact, P-bodies are considered essential for miRNA function, and the current assumption is based on the evidence that P-bodies contain separate compartments for RISC assembly and RISC recruitment of silencing proteins of both the Slicer-dependent silencing path (deadenylation enzymes Ccr4, Not1, Pop2, decapping enzymes, nucleases Xrn1p, all involved in mRNA degradation) and the Slicer-independent silencing path (p54, FMRP, Gemin5, RAP55, involved in miRNA mediated translational repression and mRNA storage) (Figure 1) [55-58].

### MicroRNAs involvement in lung cancer carcinogenesis

If we consider the numerous activities carried out by miRNAs, their involvement in cell proliferation, apoptosis, gene expression regulation and all the biological processes above mentioned, it is clear that their dysregulation may be involved in various human diseases.

Several studies have shown the involvement of miRNA deregulation and aberrant expression in the carcinogenesis of various organs, including lung cancer [59-60]. Recently, miRNAs have been classified into onco-miRNAs (Table 2) and tumor suppressor miRNAs (Table 3) in relation to their function in carcinogenic processes. Actually, different miRNAs are not yet well defined, showing both oncogenic and suppressive activities. Among them miR-7, miR-31, miR-125 and miR-183 family members were found disrupted in lung cancer [60].

**Table 2:** Dysregulated oncogenic miRNAs in lung cancer

Oncogenic miRNAs	Genomic location	Expression	Target genes
miR-21	17q23.2	Upregulated	PTEN, Spry1, Spry2, Btg2, Pdcd4, Apaf1, FasL, RhoB
miR-17-92	13q31.3	Upregulated	p21, CTGF, Tsp1, PTEN, Bim, HIF-1α
miR-221/222	Xp11.3	Upregulated	Kit, p27 kip1, PTEN/TIMP3, PUMA, TRAIL
miR-155	21q21.3	Upregulated	CASP3, TP53BP1, SOCS1, PTEN, PDC4, SHIP1
miR-494	14q32.31	Upregulated	PTEN, CASP3/7, Bim
miR-328	16q22.1	Upregulated	PRKCA, VEGF-D, NOTCH1, IL1-α, IL1-β, PLC-γ
miR-106	Xq26.2	Upregulated	RB
miR-150	19q13.33	Upregulated	TP53
miR-301	17q22	Upregulated	SMAD4, PTEN, Bim
miR-10b	2q31.1	Upregulated	HOXD10, PTEN
miR-93, miR-98, miR-197	7q22.1, Xp11.22, 1p13.3	Upregulated	FUS1

**Table 3:** Dysregulated tumor suppressive miRNAs in lung cancer

Tumor suppressive miRNAs	Genomic location	Expression	Target genes in lung cancer
let-7 family	13 members located on nine different chromosomes	Downregulated	KRAS, CDC25a, CDK6, c-MYC, CCND1, BCL-2, HMG2A
miR-143	5q32	Downregulated	c-MYC, EGFR, NUDT1, OCT4, ERK5, KRAS, MMP-13, COX-2, EMT, CD44v3
miR-145	5q32	Downregulated	c-MYC, EGFR, NUDT1, OCT4, CDK4
miR-34a, miR-34b, miR-34c	1p36.22, 11q23.1, 11q23.1	Downregulated	CDK4, CDK6, c-MYC, CCND1, CCNE2, CDC25A, MET, E2F, SIRT, AXL, SNAIL-1, PDGFRa/b
miR-449a, miR-449b, miR-449c	5q11.2	Downregulated	CDK4, CDK6, c-MYC, CCND1, CCNE2, CDC25A, MET, E2F, SIRT, AXL, SNAIL-1, PDGFRa/b
miR-15-16	13q14	Downregulated	Bcl-2, CDC2, CCND1, ETS1, JUN, MCL1, MSH2, PDCD4, PDCD6IP, RAB9B, WT1, WNT3A
miR-29a, miR-29b, miR-29c	7q32.3, 7q32.3, 1q32.2	Downregulated	DNMT3A, DNMT3B, MCL-1
miR-200a, miR-200b, miR-429	1p36.33	Downregulated	ZEB1, ZEB2, Flt1, GATA3
miR-200c and miR-141	12p13.31	Downregulated	Flt1, GATA3, KRAS, MAPK
miR-126, miR-128b	9q34.3, 2q21.3	Downregulated	VEGF, CRK, SLC7A5, EGFR
miR-133a-1, miR-133a-2, miR-133b	18q11.2, 20q13.33, 6p12.2	Downregulated	ARPC5, GSTP1, Sp1
miR-206	6p12.2	Downregulated	CCND1, GSTP1

### Oncogenic miRNAs in lung cancer

#### miR-21

Located at chromosome 17, miR-21 is one of the most studied miRNA and is the first one termed “oncomir.” It was found upregulated in different forms of solid tumors, including lung cancer [61]. MiR-21 is an anti-apoptotic miRNA and is regulated by the EGFR pathway, correlating with EGFR phosphorylated levels [62]. It has been proven that miR-21 stimulates cell growth and the invasion of NSCLC cells by targeting PTEN (Phosphatase and tensin homolog), enhancing the RAS/MEK/ERK pathway through their negative regulators repression (Spry1, Spry2, Btg2, Pdcd4) and repressing the expression of several pro-apoptotic proteins such as Apaf1, FasL, RhoB, Pdcd4 [62-65]. MiR-21 has also been found to be upregulated by KRAS in NSCLC, both *in vitro* and *in vivo* through MAPK/AP-1 activation [63-65].

#### miR-17-92 cluster

This miRNA cluster includes different members (miR-17, miR-18a, miR-19a, miR-20a, miR-19b-1, miR-92a-1), all encoded by a gene located on chromosome 13q31, and involved in several oncogenic processes like cellular proliferation, angiogenesis and apoptosis. In 2005, the Takahashi group provided evidence of their involvement in lung cancer discovering that the miRNA 17-92 cluster was strongly overexpressed in a panel of 19 lung cancer cell lines [66].

Furthermore, malignancies harboring c-MYC upregulation, such as small cell lung cancer and various B cell lymphomas, exhibit high miRNAs 17-92 levels, proving that miRNAs 17-92 cluster is under c-MYC control. In particular, miR-17-92 functions are achieved by repressing different targets involved in proliferation inhibition (p21), angiogenesis (CTGF, Tsp1) and by repressing pro-apoptotic agents (PTEN, Bim) [67]. HIF-1α is another direct target of miR-17-92, and recent evidence suggests that the induction of miR-17-92 may play a role in c-MYC mediated repression of HIF-1α [68]. However, these authors also observed that miR17- 92 repressed HIF-1α only under a normoxic condition, whereas HIF-1α was markedly induced under hypoxia, regardless of miR-17-92 levels [68]. Even though HIF-1α has been found upregulated in several cancers, probably because of the intratumoral hypoxia, its involvement in cancer initiation and progression is not clear. In fact, the ability of HIF-1α to induce cell cycle arrest by activating p21 or p27, and induce apoptosis by stabilization of p53 and transactivation of BNIP3, has been well defined [69- 70]. Similarly, HIF-1α represses c-MYC transcriptional activity and function, suggesting another role in cell cycle negative control [71]. The transcription factor E2F1 is an additional target of c-MYC that promotes cell cycle progression. O'Donnell et al. demonstrated that expression of E2F1 is negatively regulated by two miRNAs of this family, miR-17-5p and miR-20 [72]. Later, in 2007, Woods and his group proposed a model in which miR-17-92 promoted cellular proliferation through the enhancement of a proliferative E2F3 transcriptional network rather than the pro-apoptotic E2F1 activity [73]. In conclusion, two main mechanisms, which lead to an overexpression of the miR-17-92 cluster, can be observed in lung cancers: amplification of the miRNA cluster and increased expression of the c-MYC gene [66].

#### miR-221/222

MiR-221 and miR-222 are two highly homologous miRNAs encoded in tandem on the X chromosome, and involved in the development and progression of different types of epithelial cancers [77]. A growing body of evidence suggests that miR-221/222 are involved in the resistance of NSCLC cells to TRAIL (Apo2L/TNFα-related apoptosis-inducing ligand) [74], and this hypothesis are further corroborated by the observation that NSCLC cells transfection with anti-miR-221/222 results in TRAIL sensitivity in NSCLC [74]. Moreover, Kit and p27 kip1 have been identified as the main targets of this miRNA cluster in NSCLC, and the decreased levels of p27 kip1 seem to account for the reduced sensitivity to TRAIL-induced apoptosis [74]. However, additional targets of miRNAs 221-222, such as PTEN/TIMP3 [75] and PUMA (also known as Bcl-2 binding component 3) [76], seem to mediate TRAIL resistance, migration and invasiveness, thus correlating with the frequent overexpression of these miRNAs in epithelial cancers, including lung cancers [78]. Not only, but the MET oncogene is involved in miR-221/222 activation, through the JNK, AP-1 and in particular c-Jun transcription factor [75].

Additionally, miRNAs 221-222 upregulation in NSCLC is involved in tumorigenesis and aggressive biological behavior through the activation of PI3K/Akt pathway and metallopeptidases [75].

#### miR-155

Although most proof of miR-155 oncogenic roles derives from studies on lymphatic system malignancies, its involvement in cancer pathogenesis has also been confirmed in breast, liver and especially lung cancer [79].

MiR-155 oncogenetic properties depend on its antiapoptotic function, due to the blockage of caspase 3 activity and suppression of pro-apopotic genes such as TP53BP1 [80-81].

MiR-155 also promotes cell proliferation through the repression of SOCS1 [82] and by downregulating several tumor suppressors such as PTEN, PDC4 and SHIP1, leading to the activation of the Akt pathway [83]. MiR-155 is also regulated by the transforming growth factor-beta/Smad 4 pathway and plays a role in cell invasion by targeting RhoA [84].

#### miR-494, miR-27a, miR-328

miR-494 acts as an oncomir by repressing apoptosis processes targeting PTEN and caspases 3/7, whose levels are all decreased in human bronchial epithelial cells (transformed by chemical carcinogen anti-Benzo-A-Pyrene-Diol-Epoxide) harboring miR-494 upregulation [85]. Furthermore, miR-494, once activated by the ERK 1/2 pathway in NSCLC, targets BIM 3′ UTR, inducing the downregulation of BIM and consequently inhibiting apoptosis [86].

MiR-27a overexpression promotes cell growth by the suppression of the target gene FBXW7 which is involved in the malignant transformation of the human bronchial epithelial cells [87].

MiRNA 328 upregulation has recently been associated with cell migration and NSCLC brain metastases by controlling the VEGF/IL1 signaling pathway. Many of the miR-328 targets control this signaling pathway leading to loss of cell adhesion and increased migration. These target genes included PRKCA, VEGF-D, NOTCH1, IL1-alpha, IL1-beta and PLC-gamma [88].

#### miR-106, miR-150, miR-131, miR-10b

miR-106 and miR-150 were identified as oncomirs in lung cancer because of their activities in regulating growth and apoptosis. MiR-106 targets RB while miR-150 targets TP53 [89]. MiR-301 is another oncomir upregulated in several types of cancer, including NSCLC, and involved in smad4 repression through the direct binding to 3′ UTR [90-91]. Recently, also miR-10b has been reported to be overexpressed in NSCLC, playing a role in the invasion and metastasis development by HOXD10 inhibition and RhoC upregulation [92].

#### miR-93, miR-98, miR-197

These miRNAs promote lung cancer growth by repressing FUS1, a supposed tumor suppressor gene which is often downregulated in lung cancers. FUS1 works as a pro-apoptotic agent through the activation of the intrinsic mitochondrial pathway and the blockage of tyrosine kinases function, such as EGFR, PDGFR, AKT, c-Abl, c-Kit. [93-95].

### Tumor suppressive miRNAs in lung cancer

#### let-7 family

let-7 was the first miRNA identified as dysregulated in human lung cancer [96]. The let-7 miRNA family are important regulators in controlling lung cancer oncogenes expression by binding to the 3′ untranslated regions of their target mRNAs and lower levels have been found in NSCLC [97-98]. let-7 directly regulates many key cell cycle proto-oncogenes, such as KRAS, CDC25a, CDK6, c-MYC, cyclin D, BCL-2 and in this way controls cell proliferation by negatively regulating the pathways promoting the G1 to S transition [99]. One of the most interesting aspects of the let-7 family is that the 3′ UTR of KRAS (as well as HRAS, NRAS and various members of RAS GTPase family) contains multiple let-7 binding sites and that the expression of let-7 in lung cancer is inversely correlated to KRAS expression [98-100]. Moreover, Chin et al. described a novel SNP (Single Nucleotide Polymorphism) in the 3′ UTR of the KRAS gene that influences the let-7 binding to its target site. This variant, the let-7 complementary site (LCS6) in the KRAS 3′ UTR, is associated with KRAS gene upregulation and let-7 lower levels [101]; although this polymorphism correlates with a modest increase in lung cancer risk [101], other studies did not find an association between the LCS6 polymorphism and lung cancer survival, limiting its clinical usefulness in predicting an increased risk for NSCLC [102]. Another target of let-7 is HMG2A, a transcription factor involved in the activation of several genes that influence the cell growth, differentiation and proliferation. HMG2A is highly expressed in lung cancer and let-7 regulates its levels by destabilizing its mRNA through the 3′ UTR binding [103-105].

Lastly, a recent study identified HOXA1 as a new target of the let-7 family. In this study Min Zhan et al. demonstrated that let-7c represses NSCLC cell proliferation and tumorigenesis by directly targeting the 3′ UTR of HOXA1 mRNA, which subsequently reduced the expression of CCND1, CDC25A and CDK2 [106].

#### miR-143, miR-145

miR-143 and miR-145 are co-transcribed from a bicistronic gene cluster on chromosome 5, and have been identified as tumor suppressors in different types of cancer such as bladder, gastric, cervical, breast, prostate, leukemia and lung in which furthermore they are strongly downregulated [107-114]. Both miR-143 and miR-145 are able to inhibit cell growth, proliferation and migration of lung cancer cells through the repression of c-MYC, EGFR, NUDT1 and OCT4 [115-117]. In addition, miR-145 also induces cell cycle arrest in G1 by targeting CDK4 [116]. Many other relevant targets of miRNA 143 have recently been identified: ERK5, KRAS, MMP-13, COX-2 and EMT [118]. Moreover, miR-43 inhibits the migration and invasion of NSCLC by targeting CD44v3, a transmembrane glycoprotein involved in many cellular processes like the regulation of cell division, survival, migration and adhesion [118-119].

#### miR 34-449 family

The miR-34 family consists of tumor-suppressive miRNAs (miR-34a, miR-34b, miR-34c) reported to be under p53 regulation and involved in controlling apoptosis and G1 cell cycle arrest. MiR-34 reduced expression has been found in various cancers, including NSCLC [120-121]. The miR-449 cluster (miR-449a, miR-449b, miR-449c) belongs to the same family as miR-34 (p53- responsive microRNAs) [122]. Specifically, both miRNA 34 and 449 lead to cell cycle arrest, targeting CDK4, CDK6, c-MYC, CCND1, CCNE2, CDC25A, MET and E2F transcription factors family, induce apoptosis targeting Bcl-2, n-MYC, HDAC1 and upregulate p53 through the repression of deacetylase gene SIRT1 [122- 124]. miR-34 and miR-449 are also involved in the inhibition of NSCLC cell migration and invasion by suppression of AXL and SNAIL-1, respectively a tyrosine-kinase receptor and a zinc-finger protein involved in cellular migration, proliferation and cancer cell epithelial-mesenchymal transition [123-124]. Other targets identified in NCSLC are HMGA2, SERPINE1 (both implicated in cell migration and cancer invasiveness) and PDGFRa/b, whose downregulation inhibits proliferation, cell growth, and enhances TRAIL-induced apoptosis [125-126].

#### miR-15-16

miR-15 and miR-16 are important negative regulators of cell cycle progression in NSCLC and in many other solid tumors, as well as in hematologic malignancies such as chronic lymphatic leukemia [127- 128]. Located at chromosome 13q14, these miRNAs are implicated in cell cycle control and apoptosis by targeting different molecules involved in these processes (Bcl-2, CDC2, CCND1, ETS1, JUN, MCL1, MSH2, PDCD4, PDCD6IP, RAB9B, WT1, WNT3A) [128]. Furthermore, a recent study has shown that miR-15a, miR-16-1 cluster and related miR-15b, miR-16-2 cluster are direct transcriptional targets of E2F1 and control E2F-dependent cell proliferation by cyclin E gene repression, thus inhibiting the G1/S transition [129]. Another interesting observation is that in NSCLC the miR-15/16 cluster directly regulates cyclin D1, D2, E1, CDK4/6 and that cyclin D1 and miR-15/16 expression levels are inversely correlated. Moreover it has been demonstrated that, when combined, miR-34a and miR-15a/16 induce a deeper and longer lasting G1 cell cycle arrest than the repression due to only the additive effect of two miRNAs separately, suggesting a strong cooperation between them [130].

#### miR-29 family

The tumor suppressor miR-29 family includes miR-29a, miR-29b, miR-29c, and several studies have reported its downregulation in NSCLC [61, 131]. MiRNA 29 has been found to regulate DNMT3A and 3B, two DNA methyltransferases repeatedly found to be overexpressed in different kind of malignacies, including lung cancer. By targeting DNMT3A and 3B, the miRNA 29 family controls tumorigenicity both *in vivo* and *in vitro* through a demethylation process, thus leading to the re-expression of silenced tumor suppressor genes such as FHIT and WWOX [132]. Furthermore, miRNA 29 suppresses tristetraprolin (TTP) and MCL-1, respectively a protein involved in epithelial cells polarity and metastasis, and an antiapoptotic member of the Bcl-2 family [133-134].

#### miR-200 family/miR-205

The miR-200 family includes five members: miR-200a, miR-200b, miR-429, miR-200c and miR-141; in humans miR-200a, miR-200b and miR-429 co-localize at chromosome 1, while miR-200c and miR-141 at chromosome 12 [135].

Along with miR-205, this family inhibits epithelial mesenchymal transition by targeting ZEB1 and ZEB2; in lung cancer miR-200c overexpression causes a reduced expression of ZEB1 and derepression of E-cadherin, the trascriptional target of ZEB1 [136].

The Kurie group validated Flt1 as one of miR-200s targets [137]; they subsequently discovered that GATA3, which is a component of the Notch signaling pathway, was downregulated by miR-200s [138]. On the other side, Korpal et al. demonstrated that metastatic colonization to the lung was promoted by miR-200s by targeting Sec23a [139].

In addition, also KRAS was recognized as a target of miR-200c and its upregulation plays a role in overcoming chemotherapeutic treatment [140]. Furthermore, miR-200c strongly interacts with the MAPK and ERBB signaling pathway by controlling a multitude of target genes, such as the adaptor proteins Shc and Sos, but also kinases like MEKK1 and PKC or transcription factors, mainly SRF and JUN. This evidence suggests that miR-200c is a potent inhibitor of tumor progression and therapy resistance [140].

#### miR-126/126*, miR-128b

MiR-126 (or miR-126-3p) and its complement miR-126* (miR-126-5p or miR-123) are encoded by the inton 7 of Growth factor–like domain 7 (EGFL7) gene [141]. They are considered as tumor suppressor miRNAs because of their activity in decreasing lung cancer cell growth and inducing cell cycle arrest at G1 phase by targeting VEGF [141]. In NSCLC, miR-126/126* also play a role in inhibiting cell migration, adhesion invasion by targeting CRK [142], and several studies have reported their downregulation in NSCLC [143-145].

Moreover, it was demonstrated that miR-26 family is under-expressed in the majority of SCLC tumors, wheras its upregulation solws down the cellular proliferation by delaying the cells in the G1 phase and by targeting SLC7A5 [146].

MiR-128b is mapped on chromosome 3, within the 18th intron of ARPP21 gene [147] and a frequent loss of heterozygosity (LOH) [148] in NSCLC was discovered; EGFR is a direct target of miR-128b, so its reduced expression promotes carcinogenesis by the derepression of this oncogene [148].

Donzelli et al. demonstrated that in NSCLC, mutant p53R175H induces the expression of miR-128 through the transactivation of its host gene ARPP21, resulting in the increased chemoresistance of cancer cells. Indeed, miR-128 expression causes p21Waf1 upregulation through the inhibition of the transcriptional repressor E2F5 and its accumulation in the cytoplasm, a feature associated with the anti-apoptotic effect of p21waf1 [147].

#### miR-133, miR-206

MiR-133 and miR-206 have been termed “muscle specific” miRNAs since they are highly expressed in cardiac and smooth muscle tissues [149-150]. Recently, miRNAs expression signatures analysis revealed that miR-133 expression levels are significantly reduced in squamous cell carcinomas (SqCC), and it was demonstrated that its restoration causes the inhibition of cancer cell proliferation [151].

Multiple targets of miR-133 were identified, and two of them were confirmed in lung cancer cells, ARPC5 and GSTP1 [151].

In lung tumors, miR-206 expression levels were markedly lower in highly metastatic forms than in low metastatic tumors and normal lung tissues, and its upregulation causes the induction of apoptosis, the inhibition of lung cancer cell proliferation, migration and invasion [149].

### MicroRNAs in lung cancer diagnosis

Despite the progress of the diagnostic procedures, to date a population-based screening does not exist, and around the 40% of the patients with NSCLC are diagnosed in an already locally advanced or metastatic stages (IIIA/B, IV), therefore with an unresectable disease [152]. Aberle et al. have shown a 20% reduction in lung cancer mortality rates with low-dose spiral CT compared to chest radiography, in a selected older, high-risk population. Nevertheless, this study also demonstrated an elevated number of false positive results, probably due to the presence of non-pathological intraparenchimal lymph nodes or non-calcified granulomas [153]. It is therefore of primary importance to develop non-invasive confirmation tests to overcome the problem of over-diagnosis and over-treatment due to the false positive CT. Moreover, the development of minimally invasive and sensitive biomarkers could be useful also for screening and early diagnosis of lung cancers.

MiRNAs represent a class of molecules that meets these criteria. Their expression patterns in human cancers appear to be tissue-specific, and they are stable in paraffin-embedded tissues as well as in body fluids, even in plasma and serum, as opposed to mRNAs which are easily degraded by ribonucleases. These features make them potential biomarkers for cancer detection [154-156].

Yanaihara et al. in 2006 discriminated lung cancer tissues from adjacent noncancerous lung tissue using miRNA microarray analysis, and found a unique miRNA profile in lung cancer tissue, specifically a high expression of let-7a-2 and low expression of miR-155 [131]. Later, Shen et al. studied 12 miRNAs as potential plasma biomarkers. Among them, miR-21, miR-126, miR-210, and miR-486-5p showed a significant concordance between the expression levels in plasma and the corresponding tumor tissues (*P <* 0.05) yielding 86.22% sensitivity and 96.55% specificity in discriminating NSCLC patients from the healthy control group. Moreover, these four miRNAs also showed a higher sensitivity in diagnosis of lung adenocarcinomas compared with squamous cell carcinomas (91.67% vs. 82.35%, P < 0.05) [157]. Similary, Zheng et al. found that the levels of miR-155, miR-197, and miR-182 in the plasma of lung cancer patients were significantly (P < 0.001) elevated compared with control groups, and further that the simultaneous combination of these 3 miRNAs yielded 81.33% sensitivity and 86.76% specificity in distinguishing lung cancer patients from control groups [158].

Additionally, miR-30a, miR-140-3p, miR-182, miR-210, and miR-486-5p were identified to discriminate squamous cell carcinoma from non-cancerous lung tissue with an accuracy of 94.1% in a training cohort (34 patients) and 96.2% in a test cohort (26 patients) [159]. Not only, miR-1254 and miR-574-5p increased serum levels were able to discriminate early-stage NSCLC samples from controls with over 70% sensitivity and specificity, in two separate cohorts [211]. Bianchi et al. developed a miRNA signature, based on the detection of 34 microRNAs from serum, which made it possible to identify patients with early stage NSCLCs in a population of asymptomatic high-risk individuals with 80% accuracy, and also to differentiate between the malignant and the benign lesions detected by low-dose TC. [212]. In accordance with Bianchi F., Boeri et al. validated a 13 miRNAs diagnostic signature of NSCLC that can differentiate aggressive from indolent tumors detected by low-dose TC with almost 80% accuracy. Interestingly, they showed that this signature appears months before NSCLC can be diagnosed by low-dose TC, especially in patients whose tumors had aggressive features and behavior [160]. Moreover, Barshac et. al identified different miRNA expression profiles between primary lung tumors and metastases, in particular miRNA-182 and miRNA-126 overexpression was found respectively in primary lung cancers and in metastatic tumors [161].

Since it is the most easily accessible biological fluid, different studies evaluated the possibility of miRNAs detection in sputum. Ying Xie et al. examined miRNA 21 and miRNA155 expression by real-time reverse transcription polymerase chain reaction (RT-PCR) in sputum of 23 patients with NSCLC and 17 cancer-free subjects. MiR-21 expression in the sputum specimens was significantly higher in patients with NSCLC than cancer-free control group (p<0.0001) [162]. Yu et al. found that the combined overexpression of miR-21, miR-200b, miR-375, and miR-486 in surgical tissues and sputum were biomarkers for lung adenocarcinoma diagnosis, with 80.6% sensitivity and 91.7% specificity [163]. Similarly, Xing L. et al. developed a sputum-based biomarker panel based on three miRNAs (miR-205, miR-210 and miR-708) for the diagnosis of squamous cell lung cancer, yielding a sensitivity of 73% and a specificity of 96% [164]. Finally, Roa WH. et al. optimized a five miRNAs panel (miR-21, miR-143, miR-155, miR-210, miR-372) for early NSCLC detection, with a 83.3% sensitivity and 100% specificity [165].

An ulterior feature of the use of microRNAs for diagnostic purposes, is the ability to discriminate between different histological types. Lebanony D et al. validated a microRNA-based qRT-PCR assay that measures the expression levels of miR-205, reaching a sensitivity of 96% and specificity of 90% in the identification of squamous cell lung carcinomas [166]. Similar results were found by Boeri et al., confirming the role of miR-205 and miR-21 expressions in discriminating between adenocarcinoma and squamous cell carcinoma [160]. Additionally, Bishop et al. showed a 100% concordance between the diagnoses established by conventional (histologic features and immunohistochemical profiles) and miRNA-based methods (quantitative reverse transcription-PCR diagnostic assay), using miRNA 205 overexpression as a positive biomarker for squamous cell carcinoma [167]. Landi et al. identified a five-miRNA signature (miR-25, miR-34c-5p, miR-191, let-7e, miR-34a) that strongly differentiated squamous cell carcinoma from adenocarcinoma (global P< 0.0001), and reported that the lower expression level of this signature correlated with the poor overall survival rates of squamous cell carcinoma patients [168].

In summary, all of this evidence shows that miRNAs are useful biomarkers to differentiate lung cancer tissue from healthy tissue, and specifically how they can discriminate between the different histological types of lung cancer, or between lung primary tumors and metastases. Moreover, as mentioned above, the possibility of their detection in sputum specimens could be particularly helpful in early lung cancer diagnosis.

### Prognostic role of miRNAs in lung cancer

In 2004, Takamizawa et al. reported that reduced let-7 expression was significantly associated with shortened postoperative survival in patients affected by NSCLC, suggesting the potential value of miRNAs in defining lung cancer prognosis for the first time [96]. Similar results were obtained by Yanaihara et al., who showed that overexpression of the precursor of miR-155 and the downregulation of let-7a-2 correlated with poor survival in NSCLC patients [131]. Yu et al. validated a further miRNA signature based on five miRNAs, including let-7 (let-7a, miR-137, miR-182, miR-221, miR-372). They found that patients with high-risk scores in their microRNA signatures had poor overall and disease-free survivals compared to the low-risk score patients [169]. In this respect, Heegaard et al. studied paired serum and plasma samples from 220 patients with early stage NSCLC and 220 controls, using qRT-PCR to measure 30 different circulating miRNAs. The expression of miR-146b, miR-221, let-7a, miR-155, miR-17-5p, miR-27a and miR-106a was significantly reduced in the serum of patients with NSCLC and miR-29c was significantly increased, while low levels of plasma-miRNA let-7b were statistically significantly associated with poor survival [170]. Xiaogang Tan et al. developed a further 5 five miRNAs signature that could distinguish SCC from normal lung tissues, and demonstrated that high expression of miRNA-31 was associated with poor survival in stage I-III squamous cell lung carcinoma [159]. MiR-31 was also validated as a marker for lymph node metastasis as well as a predictor for survival outcomes in patients with lung adenocarcinoma [171].

Lately, many other miRNAs have been studied as possible prognostic factors, in particular Raponi et al. reported that high expression of miR-146b correlated with a poor overall survival in patients undergoing radical surgery for squamous cell carcinoma, also experiencing the same trend in the expression level of miR-155 in the same group of patients [172]. Nevertheless, Wu et al. showed that high miR-19b and low miR-146a expression in NSCLC tissues were associated with higher TNM stage, lymph node metastasis and poorer survival (P < 0.05) [173].

Furthermore, Li et al. detected the expression of miR-146 in NSCLC tissues and normal tissues by MISH (miRNA in situ hybridization), concluding that high expression level of this miRNA can predict NSCLC prognosis [174].

Recently, the possible prognostic role of miR-155 has been also explored, and several studies confirm its value in lung cancer diagnosis, prognosis and therapy [79]. In this regard, Donnem et al. evaluated the prognostic impact of miR-155 overexpression in 91 squamous cell carcinomas, 95 adenocarcinomas, 31 large cell carcinomas and 18 bronchioalveolar carcinomas by in situ hybridization. They found that in patients affected by adenocarcinoma, high miR-155 expression levels tended to assume a negative prognostic significance on survival in univariate analysis (P = 0.086), and were also an independent prognostic factor in multivariate analysis (HR 1.87, CI 95% 1.01 - 3.48, P = 0.047). In the group of squamous cell carcinomas with lymph node metastasis, miR-155 had a positive prognostic impact on survival in both univariate (P = 0.034) and multivariate analysis (HR 0.45, CI 95% 0.21-0.96, P = 0.039). These data suggest that the prognostic role of miRNA 155 could be different according to the histological subtype and nodal status [175]. Despite this, high miR-155 expression levels were found to be associated with poor outcome in lung squamous cell carcinoma [172]. Nonetheless, Tong-Peng Xu et al. conducted a meta-analysis of 15 published articles including 2,463 subjects. They demonstrated that patients with high miR-155 expression generally had a poor outcome, while stratified analysis showed that miR-155 overexpression was not statistically correlated to overall survival in lung cancer; on the other hand, when the hazard ratio for recurrence free survival (RFS) and cancer specific survival (CSS) was combined, the increased expression of miR-155 was significantly associated with RFS as well as CSS [176]. Therefore, the role of miR-155 in lung cancer prognosis requires further investigation.

Also, miR-126 family received particular attention for its role in lung cancer pathogenesis, especially for the ability to regulate angiogenesis through VEGF-A release from the tumor cells. Donnem T. et al. showed that miR-126 overexpression was a significant negative prognostic marker in SCCs (P < 001) and in lymph node positive patients [177].

Various studies evaluated also the prognostic role of high miR-21 levels. Markou A. et al. demonstrated for the first time that mature miR-21 overexpression, achieved using qRT-PCR, correlated with poor overall survival (OS) (P = 0.027) [178]. Gao W. et al. found that high miRNA-21 and low 181 miRNA levels were associated with poor survival, independent of clinical covariates, including TNM staging and lymph node status [179]. In a further study, the same investigators showed that the high-level expression of miR-21 in human primary squamous cell lung carcinoma was significantly correlated to shortened survival time [143]. Liu XG et al. confirmed that high expression of serum miR-21 and tumor miR-200c are associated with poor prognosis in lung cancer patients [180]. These findings were confirmed and expanded upon by many other studies [181-183], in particular Voortman et al., who determined the expression of miR-21, miR-29b, miR-34a/b/c, miR-155 and let-7a, by quantitative real-time PCR in paraffin embedded formalin fixed tumor specimens from 639 patients enrolled in the International Adjuvant Lung Cancer Trial. Surprisingly, only the low expression of miR-21 was associated with shorter survival time [182].

Several other miRNAs have been investigated individually because of their potential role in the prediction of the lung cancer prognosis, in particular low expression levels of miR-34a, miR-374a, miR-181a, miR-221, miR-200c and miR-218 were reported to associate with a poor survival or high risk recurrence in lung cancers [60].

Arora et al. found miR-328 to be a marker for patients with higher risk for brain metastases. In this study, both PRKCA and IL1-beta were identified as targets of miR-328, supporting evidence that miR-328 leads to increased migration of NSCLC cells, through this pathway as well [89]. Similarly, miRNA-145 downregulation was found to play various important roles in the development of brain metastases from lung adenocarcinoma [184]. Finally, the latest evidence indicates that epigenetic changes in miRNA expression may contribute to defining the prognosis of patients with NSCLC. Specifically, Wang Z. et al. demonstrated that patients affected by NSCLC and underwent curative surgery (without receiving any subsequent adjuvant therapy) with aberrant DNA methylation of miR-34b/c (but not 34a), had a high probability of recurrence, poor overall survival and poor disease-free survival [185]. Again, Gallardo et al. found a correlation between MIRN34A gene promoter region methylation and miR-34a expression (P = 0.008) [186]. They also found that the frequency of p53 mutations was significantly higher in patients with low miR-34a expression, and the group of patients harboring both p53 mutations and low miR-34a expression had a poor prognosis [186]. The same role of miR-34b/c aberrant methylation was reported even in SCLC [187]. Kitano et al. analyzed the methylation status of miR-152, miR-9- 3, miR-124-1, miR-124-2, and miR-124-3 in 96 NSCLC specimens, discovering that methylation of miR-9-3, miR-124-2, and miR-124-3 were individually associated with an advanced T-factor independent of other clinical covariates, such as age, sex, and smoking habit. They also found that methylation of multiple microRNA loci was associated with a poorer progression-free survival rate in univariate analysis [188]. Not only, but the let-7 miRNA gene family and was found to be hypomethylated in lung adenocarcinomas, while normally methylated in normal human tissues by DNA methyltransferases DNMT1 and DNMT3B [189]. Further studies have also confirmed that miR-1 and miR-29 family are subject to epigenetic regulation, prospecting a possible pathogenetic as well as prognostic and therapeutic role [190-191].

Just like epigenetic changes, single-nucleotide polymorphisms (SNP) in miRNAs genes are also involved in lung cancer susceptibility and prognosis [192-193]. Tian Tian et al. showed that the SNP rs11614913 T>C in the pre-miRNA region of miR-196a2 was related with significantly increased risk and poor survival among Chinese patients affected by NSCLC [194]. It should be noted that when the role of miR 196a in the pathogenesis and prediction of patients with NSCLC outcome was recently confirmed, it was found that miR-196a is significantly upregulated in NSCLC tissues, and that it is involved in NSCLC cell proliferation, migration and invasion, mainly via the downregulation of HOXA5. Higher expression of miR-196a in NSCLC tissues was also associated with higher clinical stages and lymph-node metastases [195].

Naturally, SNPs also occur in miRNA target genes and miRNA processing machinery genes. In particular, a SNP in the let-7 binding site at KRAS 3′ UTR (LSC6) enhances KRAS expression levels, resulting in increased risk of NSCLC in patients who are moderate smokers [101]. Similarly, Xiong F. et al. identified a SNP in the 3′- UTR of the L-MYC gene MYCL1 that results in increased susceptibility to SCLC, probably due to a defective interaction between miR-1827 and its complementary MYCL1 3′-UTR binding site [196]. Finally, also SNPs in miRNA processing machinery genes such as Drosha, AGO1, XPO5 were reported associated with lung cancer prognosis, and in the near future may contribute to a further stratification of these patients [197-200].

### Predictive role of miRNAs in lung cancer

Lung cancer therapy has changed enormously over the last years thanks to the discovery of specific mutations (EGFR, KRAS, ALK, ROS1), and thus the introduction of targeted therapies. Certainly, the understanding of the mechanisms of resistance that often affect the outcome of these patients is equally important. The study of the predictive role of miRNAs is still a relatively unexplored frontier that is already of great importance.

Weiss et al. demonstrated that miR-128b directly regulates EGFR and that a loss of heterozygosity in miR-128b was significantly associated with survival benefit in patients treated with gefitinib [148]. Similarly Zhong et al. showed that miR-126 restoration enhances gefitinib-induced cytotoxicity in lung cancer cells [201], while Cho et al. found that the restoration of miR-145 inhibits cancer cell growth in lung adenocarcinoma patients with EGFR-activating mutations [202]. Moreover, miR-21 is positively regulated by EGFR signaling in cancer cells harboring activating EGFR mutations, and EGFR-TKIs can repress the aberrantly increased miR-21 levels, while miR-21 suppression could enhance EGFR TKIs therapeutic effects [203]. In addition, miR-21 was found to be involved in the acquired resistance of EGFR-TKI in NSCLC (*in vivo* and *in vitro*), through the downregulation of PTEN, PDCD4 and by the activation PI3K/Akt pathway [204]. Another study showed that miR-7 downregulates EGFR mRNA in different cancer cell lines, including lung cancer. This study confirmed that miR-7 negatively regulates also AKT, and ERK 1/2 [205], whereas Rai et al. also confirmed these results finding that miR-7 ectopic expression allowed EGFR TKIs to overcome resistance in lung cancer cell lines [206].

Noteworthy is the finding that some miRNAs are able to inhibit TRAIL-induced apoptosis in lung cancer cells. TRAIL (TNF-related apoptosis inducing Ligand) is a member of the TNF family involved in programmed cell death in cancer cells [207], and miR-221, miR-222 were found to guide lung cancer resistance to TRAIL therapy by downregulating PTEN and TIMP3, which are both tumor suppressors [75].

The evidence that miR-628 overexpression is associated with resistance to crizotinib treatment, since crizotinib-induced cell death occurs through activation of the caspase-3, which is a miR-628 target, is also noteworthy [208-209].

In the case of traditional chemotherapy, mir-181a sensitized A549 cells to the lethal action of cisplatin by stimulating Bax oligomerization as well as through the activation of proapoptotic caspases 9 and 3 [210]. MiR-630, on the other hand, arrests the main manifestations of the DNA damage response to cisplatin such as ATM, p53 and histone H2AX phosphorylation, leading to G0- G1 phase cell cycle arrest and a diminished sensitivity of A549 cells to S-G2-M cell cycle arrest [210]. Additionally, miR-17-92 cluster and miR-221/222 were found to enhance lung cancer cell sensitivity to cytotoxic drugs [66, 59, 75]. Furthermore, Gao et al. investigated the role of miR-21 in predicting adjuvant platinum-based chemotherapy response and disease free survival in patients with NSCLC. They found that high levels of miR-21 increased A549 cells resistance to platinum, while reduced miR-21 decreased the resistance of A549 cells to platinum. This data was confirmed in the tissue samples of 58 patients, in which miR-21 expression was significantly increased in platinum-based chemotherapy resistant patients (n=58, P=0.000) and also associated to a shorter disease free survival (P = 0.008) [213]. Similar findings about miR-503were reported by Qui T. et al., indeed they showed that the expression of miR-503 was decreased in cisplatin-resistant NSCLC cells (A549/CDDP), compared with the parental A549 cells, and that the overexpression of miR-503 could sensitize the A549/CDDP cells to cisplatin targeting the anti-apoptotic protein Bcl-2, whereas the inhibition of miR-503 was able to increase A549 cell resistance to cisplatin [214]. MiR-135a/b overexpression was found to reduce MCL1 protein levels and sensitize A549 cells to cispatin-induced apoptosis, suggesting a potential role of miR-135a/b loss of function in the development of cisplatin resistance in lung cancer [215].

Finally, miR-101 was discovered to enhance paclitaxel-induced apoptosis in NSCLC cells by the repression of EZH2 [216] and sensitize the A549 NSCLC cell line to cisplatin-induced apoptosis through the caspase 3 pathway [217].

MiRNAs have also been examined for their role in predicting the response of radiation therapy. In particular, let-7b downregulation lead to an important protection from radiation, conversely let-7g downregulation increased radiosensitization in lung cancer cells (A549) while its overexpression can act as protection against radiation damage [218]. As previously reported, miR-21 is often overexpressed in NSCLC, also associated with lymph node metastasis and poor prognosis. Recent data suggests its involvement in the radio-resistance of lung cancer cells by silencing the expression of crucial genes implicated in apoptosis through caspase activation [219].

In conclusion, there is a body of evidence to support the idea that miRNAs may have a predictive role in defining response to both biological treatments and traditional chemotherapy. Further studies are required to confirm and extend this data in order to contribute to a better stratification of patients and thus develop personalized therapeutic strategies.

### MiRNAs in lung cancer therapy

The possibility of using miRNAs for therapeutic purposes is one of the most interesting innovations in lung cancer therapy.

Recently, several studies on mice models of NSCLC paved the way for “miRNA replacement therapy” using exogenous delivery of let-7 to reduce tumor growth, showing a significative effect [220-221]. Similarly, let-7 double stranded mimics were found able to inhibit cellular growth and migration as well to induce the cell cycle arrest of lung cancer cell lines *in vitro* [222]. Wiggins et al. used chemically synthesized miR-34a and a lipid-based delivery vehicle to reduce tumor growth in mouse models of NSCLC, confirming the hypothesis that the reintroduction of lost miRNAs in lung cancer cells reactivates the physiological inhibitory pathways involved in cancer control [223]. Interestingly, Trang et al. used synthetic tumor suppressors miR-34 and let-7 mimics complexed with a novel neutral lipid emulsion to target a KRAS-activated mouse model of NSCLC. Systemic delivery of these miRNA mimics led to a significant decrease in tumor burden, providing a confirmation of the promising role of miRNA mimics in lung cancer therapy [224]. Moreover, the observation that miR-145 is able to inhibit cell growth and the G1/S transition in transfection assays of A549 and H23 cells by targeting c-MYC, provide an innovative approach to the treatment of NSCLC [116]. Regarding the resistance to TKIs treatment that may affect several patients, miRNA delivery systems could play a key role in overcoming this resistance. Rai et al. used cationic liposomes loaded with miR-7–expressing plasmid to inhibit EGFR signaling causing a dramatic response in an EGFR-TKI–resistant lung cancer xenograft model [225]. Zhao et al., on the other hand, demonstrated that miR-34a mimics augment the sensitivity to erlotinib treatment in NSCLC and HCC cell lines, whether they were associated with primary or acquired resistance [226]. These findings are supported by the evidence that miR-34a targets MET and AXL, both involved in erlotinib resistance [227-228]. This data suggests a potential role of erlotinib-miR34a combination therapy, while a phase I clinical trial based on MRX34, a liposomal nanoparticle loaded with synthetic miR-34a mimics, was recently initiated [229].

On the other side, oncomir targeting therapies have been developed in recent years with promising results. “Antagomirs” and “LNA-antimiRs” represent new classes of antisense oligonucleotide with specific chemical modifications such as 2′-O methyl and Locked Nucleic Acid (LNA), which make them less susceptible to nuclease enzymes cleavage. These kinds of molecules could play a considerable role in miRNA silencing processes, thus suppressing those miRNAs with oncogenic properties [230-231]. Li et al. used an anti-miR-150 expression vector to suppress A549 cell proliferation by targeting miR-150. Tumor volume and weight were lower compared with the control group [232].

Elmen et al. showed that the administration of LNA-antimiR was able to silence miR-122 through the formation of stable heteroduplexes between the LNA-antimiR and miR-122 in primate hepatocytes, confirming their potential role in miRNAs targeted therapies [231]. However, the ability to negatively regulate miRNAs levels had already been explored with success by Krützfeldt et al. in 2005. They showed that intravenous administration of antagomirs against miR-16, miR-122, miR-192 and miR-194 resulted in a significant reduction of corresponding miRNA levels in different tissues and organs like liver, lung, kidney, heart, intestine, fat, skin, bone marrow, muscle, ovaries and adrenals [230].

Ebert et al. introduced another innovative method to induce loss of function in miRNAs by developing “miRNA-sponges”, a novel class of competitive inhibitors of small RNAs in mammalian cells. Produced from transgenes within cells, sponge-miRNAs contain complementary binding sites to a miRNAs seed region, permitting a deep inhibition of whole classes of related miRNA which harbor the same seed site [233-234]. More recently, Obad et al. developed a further method to target the miRNA seed region, based on seed-targeting 8-mer locked nucleic acid (LNA) oligonucleotides, named “tiny LNAs” [235].

In conclusion, lipid-formulated mimics, antagomirs, miRNA sponges, virus vectors, locked nucleic acids (LNAs) as well as antisense oligonucleotides, all represent new promising therapeutic approaches based on the knowledge and manipulation of miRNAs, providing an important new tool in personalized therapy for lung cancer.

### Small interfering RNAs in lung cancer

Small interfering RNAs (siRNA) represent a 19- 23 nucleotides class of small non-coding RNA involved in post-transcriptional gene silencing through the RISC-mediated degradation mechanism of mRNA targets. Produced from long dsRNAs of exogenous or endogenous origin by a ribonuclease-III type endonuclease (DICER), they are made up of two partially complementary RNA single strands, named passenger strand and guide strand. SiRNAs are loaded into Argonaute 2 (Ago2) and the guide strand is subsequently incorporated in the RNA-induced silencing complex (RISC), leading to a sequence-specific degradation of complementary target mRNAs [27].

**Figure 2:**
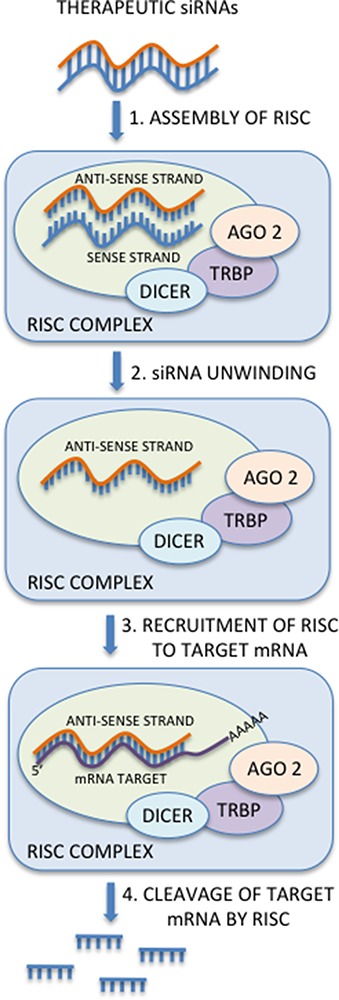
SiRNAs mechanism of action The RNA-interference mediated knockdown of gene expression in mammalian cells is based on the introduction of synthetic double-stranded siRNAs or plasmid and viral vector systems expressing double-stranded short hairpin RNAs (shRNAs) that are processed by the cellular machinery into siRNAs. Once loaded into Argonaute 2 (Ago2), the guide strand is incorporated in the RNA-induced silencing complex (RISC), leading to a sequence-specific degradation of complementary target mRNAs.

Since their discovery, siRNAs have shown a strong role in regulating gene expression, being able to silence target genes in a highly specific manner. Because of these characteristics, siRNAs were immediately confirmed as indispensable tools for gene silencing experiments, and for this reason the possibility of their use in cancer therapy has been studied with promising results. The RNA-interference mediated knockdown of gene expression in mammalian cells is based on the introduction of synthetic double-stranded siRNAs or plasmid and viral vector systems expressing double-stranded short hairpin RNAs (shRNAs) that are processed by the cellular machinery into siRNAs (Figure 2).

Matsubara et al. evaluated the antitumor effects of mTOR-siRNA therapy in NSCLC cells, showing an increased level of apoptosis (P = 0.016), as well as a deep inhibition of cell migration (P = 0.0001) and proliferation (P = 0.034) [236]. Furthermore, Xu et al. designed a specific small interfering RNA to target tissue factor (TF), a transmembrane glycoprotein recently discovered to be involved in lung cancer tumorigenesis. They demonstrated a dose-dependent downregulation of TF both *in vitro* and *in vivo*, leading to the inhibition of proliferation, invasion, metastasis, and to the induction of apoptosis, also resulting in the MAPK-ERK, PI3K/Akt and VEGF pathways suppression [237]. Moreover, Wu et al. identified CLEF1 as a potential target of siRNA-based therapy. CLEF1 is a post-transcriptional regulator of gene expression involved in different types of cancer, including lung cancer, and whose levels are significantly higher in neoplastic tissues. In this study, CELF1 silencing by siRNA lentiviral-mediated transfection strongly reduced survival rates in lung cancer cells, likewise the number and size of lung cancer cell colonies [238].

In addition, many other possible therapeutic targets for treating lung cancer have recently been identified by siRNA technology. In detail, lentivirus-mediated siRNA knockdown of ZEB-1, NUPR1 and SGO-1 inhibits human NSCLC cell growth *in vitro* and *in vivo* [239-241].

Besides, in light of the role played by VEGF family and its receptors in the pathogenesis and especially in NSCLC target therapy, the possibility to silence the expression of these genes will surely result in a therapeutic evolution of extreme importance. In this regard Yang Y. et al. explored the antitumor efficacy of VEGF-shRNA on A549 cells *in vitro* and on A549 lung carcinoma xenografts in nude mice. VEGF-shRNA delayed tumor growth and significantly reduced tumor weight compared with controls (P < 0.05), also showing a significant angiogenesis inhibition (P < 0.01) and apoptosis induction (P < 0.01) [242]. Feng Y. et al. developed a lentivirus-mediated RNA interference to knockdown the expression of VEGF-C in A549 cells, demonstrating a suppression of cell growth, migration and invasion *in vitro*. Interestingly, these authors found that silencing VEGF-C also resulted in an overthrow of VEGFR-2, VEGFR-3, CXCR4, CCR7 signaling, and in a downregulation of Akt, ERK and p38 pathways [243]. A further study reported that the local injection of lentivirus-delivered shRNA against Livin (novel member of IAP family) into xenograft tumors derived from the lung adenocarcinoma cell line SPC-A-1 in BALB/C nude mice, induced apoptosis and reduced tumor cell proliferation, tumor growth and weight. These findings supported the evidence that Livin shRNA-mediated downregulation lead to G0/G1-phase cell cycle arrest, through cyclin D1 suppression, providing a new therapeutic approach for lung cancer therapy [244]. Li et al. evaluated the efficacy of the dual inhibition of FAK and EGFR by using plasmid vector-based RNA interference in A549 lung cancer cells, *in vitro* and in A549 subcutaneous xenograft mice model. They found that knockdown of FAK and EGFR expression *in vitro* significantly inhibited cell proliferation and induced cell apoptosis whereas mice treated with FAK and EGFR shRNA had smaller tumors (P<0.01) compared to controls, also resulting in a decreased microvessel density and cell proliferation [245]. In regards to EGFR gene silencing strategies, in 2005 Zhang et al. reported the potential role of a dsRNA-mediated specific RNAi approach for silencing the EGFR in NSCLC cell lines *in vitro* and *in vivo*. For the first time, they demonstrated a significant silencing of an endogenous cellular gene and growth inhibition *in vivo*. In addition, these authors showed that this approach enhanced the chemosensitivity to cisplatin, paving the way for an original method to overcome cisplatin resistance that may develop because of EGFR pathway iperactivation [246]. More recently, Takahashi et al. developed an interesting way to inhibit the oncogenic EGFR alleles without affecting the normal EGFR allele, both *in vivo* and *in vitro*, through allele specific RNAi (ASP-RNAi) treatment. They confirmed that the specific inhibition of the EGFR mutated allele, represents a safe and effective progress for cancer therapy, also because ASP-RNAi treatment can suppress cancer cell proliferation and growth, regardless of sensitivity to EGFR-TKIs [247].

Furthermore, Chen et al. showed that the knockdown of T790M transcript by siRNAs, recovered the sensitivity of T790M mutant cells to TKIs, decreasing cell growth and inducing apoptosis of T790M mutant NSCLC cell line H1975 treated with TKIs, or cetuximab [248]. The same authors also described that the addition of EGFR siRNA to either TKIs or cetuximab additively enhanced growth inhibition, as well as the induction of apoptosis in all cell lines tested (HCC827, H292, H358, H1650, H1975), independent of their EGFR mutation status. Also in this case the greatest biological impact was observed when afatinib was combined with EGFR-specific siRNA [249].

Given the emerging role of KRAS mutations in lung cancer oncogenesis and progression, the chance of silencing this oncogene is of vital interest. In 2006, Zhang et al. evaluated the inhibitory effects of adenovirus-mediated siRNA against mutant KRAS on NSCLCs, both *in vivo* and vitro, demonstrating a significant growth inhibition of lung adenocarcinomas [250]. Similarly, a more recent study found that mutant KRAS shRNA-knockdown in NSCLC suppresses tumor growth also sensitizing tumor cells to p38 and EGFR inhibitors [251].

Another alternative approach to targeting KRAS-driven lung cancers through RNA interference technology, is to identify and consequently silence the gene products whose inhibition may lead to cell death only in the presence of KRAS mutations. Among the potential synthetic lethal interactors investigated, the suppression of TANK-binding kinase 1 (TBK1) induces apoptosis only in KRAS mutated cancer cell lines via NFκB pathway activation [252], while Syk and Ron kinases and integrin beta6 depletion were able to induce epithelial-mesenchymal transformation (EMT) and apoptosis specifically in KRAS-dependent cells, both in lung and pancreatic cancer [253]. Remarkably, analogous findings were also reported in colorectal cancer cells harboring KRAS mutations. Here too, using a pool-based shRNA platform in a genome-wide screen, different KRAS synthetic lethal (RSL) genes were identified as possible therapeutic targets [254].

Finally, EZH2 silencing with siRNA has been proven to induce cell cycle G2/M arrest in human lung cancer cells, enhancing p53 and p21 expression and subsequently decreasing Cdc2 and cyclin B1 levels [255]. Another study explored the antitumor efficacy of siRNA-EZH2 in combination with radiotherapy *in vitro* and *in vivo*, and observed an increased inhibition of cell proliferation and cell cycle progression when the radiotherapy was associated with siRNA-EZH2 compared with radiotherapy alone [256]. Taken together, EZH2 gene silencing experiments showed promising results *in vivo* and *in vitro*, which in the near future could drive a targeted therapy based precisely on RNAi.

Despite the initial enthusiasm, several unsolved issues are still hindering the development of effective therapies based on gene silencing induced by RNAi: increase the stability of siRNA in the bloodstream, hit selective tumor tissue targets, increase the intracellular uptake and create a delivery system that ensures adequate dosing and distribution. These are currently the main challenges. To date, several types of delivery systems have been developed, viral vectors, liposome-based delivery systems, nanoparticle-based delivery systems, dendrimer-based delivery systems, carbon nanotube-based delivery systems, each of which have their own advantages and disadvantages [257]. Still, several of them are now in preclinical and clinical studies, with applications in various types of neoplasm, including NSCLC [258-260].

### Small nucleolar RNAs in lung cancer

Small nucleolar RNAs consist of a 60–300 nucleotides long class of small non-coding RNAs involved in various and essential functions, including modification, maturation and maintenance of rRNA stability, in order to ensure a correct ribosomal biogenesis and function [261]. Currently they are classified into two categories, Box C/D and Box H/ACA, on the basis of the presence of a different consensus sequence [262]. Further evidence suggests that the C/D box snoRNAs have a preminent role in the 2′-O-methylation of rRNAs whereas the H/ACA box snoRNAs are mainly involved in pseudouridylation of rRNAs [263].

Small nucleolar RNAs are located within introns of protein-coding genes transcribed by RNA polymerase II, as well as introns of long non-coding RNAs [261- 265]. C/D box snoRNA matures along either one of two different pathways. The first one involves the splicing of a pre-mRNA and subsequently the formation of a snoRNA-containing lariat, which is then linearized and cleaved by endonucleases and exonucleases, releasing the final mature snoRNA. The second maturation pathway is splicing-independent, and snoRNAs are excided from the intron regions of the pre-mRNAs by endonucleolytic cleavage [266].

However, in spite of the functions traditionally performed by the snoRNA, in 2011 Brameier et al. have demonstrated that some human snoRNAs act similarly to miRNAs. These sno-miRNAs originate from relatively short snoRNAs, showing several silencing features typical of miRNAs, appear involved in numerous cellular processes, including gene expression [267]. To date many studies have demonstrated the role the disruption of snoRNAs in several types of cancer, such as breast, prostate, B-cell lymphoma and recently acute promyelocytic leukemia [27]. Additionally, a growing body of evidence suggests a possible role of snoRNAs also in lung cancer.

Liao et al. profiled snoRNA expression signatures of early stage NSCLC by performing microarray analysis on surgical tissues, identifying six snoRNAs which, compared to noncancerous lung tissues, were overexpressed in tumor tissues. Interestingly, these authors demonstrated that snoRNAs were detectable in plasma, and three of them (SNORD33, SNORD66, SNORD76) showed higher plasma expressions in NSCLC patients compared with healthy control groups (P = 0.01), yielding a 81.1% sensitivity and 95.8% specificity in discriminating NSCLC from cancer-free individuals and patients affected by COPD [268]. This data suggests that SNORD66, SNORD76 may act like oncogenes in lung cancer, as they are located in two of the most amplified chromosomal segments in solid neoplasm (19q13.3 and 1q25.1), while SNORD33 is encoded by chromosome 19q13.3 which contains different oncogenes involved in lung cancer and other solid tumors [269-272]. In a further study, Mei et al. found that snoRNA42 was overexpressed in NSCLC cells, and demonstrated that snoRNA42 knockdown decreased tumorigenicity *in vitro* and *in vivo* by inducing p53- mediated apoptosis, whereas its upregulation promoted the cell growth of bronchial epitheliums [273]. Moreover, they observed an inverse correlation between SNORA42 expression in lung tumor tissue specimens and NSCLC patients survival [273].

Certainly, in the light of the new roles of snRNA in regulating gene expression and silencing processes, as well as cellular proliferation, differentiation and survival, their future use for therapeutic purposes appears highly topical. For instance, snoRA42 knockdown by siRNA has antiproliferative effects on NCSLC cells, leading to a diminished *in vitro* tumorigenicity. Similarly, snoRA42 knockdown was found to decrease *in vivo* tumorigenicity in both ectopic and orthotopic NSCLC xenograft mouse models [273].

However, as mentioned previously, some technical problems such as a definition of the appropriate dosage regimens and the creation of a suitable delivery system, limit the therapeutic use of siRNA.

Nevertheless, the study of the biological aspects of snoRNAs in cancer should be encouraged, and snoRNAs diagnostic and prognostic biomarkers should be developed, providing us with a new and useful tool for cancer detection and treatment.

### Piwi proteins associated RNAs in lung cancer

Piwi proteins associated RNAs (piRNAs) represent the latest identified class of small non-coding RNA, and recent evidence highlighted their role in the preservation of genomic integrity [274].

Their name derives from their ability to interact only with PIWI proteins, a subfamily of Argonaute proteins which is involved in gene-silencing pathways, whereas siRNAs and miRNAs associate with AGO subfamily members [275]. PiRNAs are 24–32 nucleotides long ncRNAs, and are generated by a Dicer-independent mechanism [276].

Currently, both in Drosophila and in mammals, two different pathways have been described in piRNAs biogenesis, a primary pathway and a secondary pathway, the latter also termed the “ping pong” model.

In the primary pathway, piRNA are transcribed from pericentromeric or subtelomeric domains, which contain transposable DNA elements, or else from 3′ UTRs of protein coding genes, and subsequently shortened into piRNA-like small RNAs [276]. The piRNAs are then exported to the cytoplasm where they undergo a maturation process which involves the putative RNA helicase Armitage (ARMI), the nuclease Zucchini (ZUC), and YB. Once 5′ ends are formed, piRNAs are loaded onto PIWI proteins, then trimmed from the 3′ and 29-O-methylated by HEN1/Pimet to produce mature piRNAs [277-278]. Unlike the primary pathway, which is shared both in germ and in somatic cells, the amplification “ping pong” cycle appears to be restricted to germ cells. In 2007, Brennecke et al. observed that AUB or PIWI associated with the antisense piRNA, cleaves the sense retrotransposon transcripts, leading to the creation of the 5′ ends of sense piRNA that in turn associates with AGO3 [276]. Then, AGO3-piRNA complex cleaves antisense retrotransposon transcripts making the 5′ end of antisense piRNAs that subsequently bind again to AUB or PIWI, establishing an amplification cycle which is mainly involved in targeting active transposons, thus safeguarding the genome [276, 279]. Nonetheless, recent studies on Drosophila demonstrated that piRNAs-PIWI complex is involved in gene silencing by binding its genomic target in euchromatin, and recruiting some important epigenetic regulators such as HP1a and the histone methyltransferase Su(var)3-9. Through a parallel mechanism, Piwi targets nascent transcripts via piRNA sequence complementarity [280].

Although most of the evidence about biogenesis and the function of piRNAs and PIWI proteins are related to studies done on Drosophila, it has been shown that this family of proteins is actually highly conserved across various species and organisms [281], and to date four PIWI proteins have been found in humans: PIWIL1/HIWI, PIWIL2/HILI, PIWIL3, and PIWIL4/HIWI2. However, in addition to the known functions performed in germ cells, it is now believed that piRNAs fulfill many other functions, as stem cell self-renewal, retrotransposon silencing, genetic and epigenetic regulation, and recently also the regulation of tumorigenesis [281]. Indeed, a growing body of evidence emphasizes the role of both piRNA and PIWI proteins in the biology of different types of cancer, including gastric, colon, lung, and breast cancer [282]. Besides, HIWI over-expression has also been found in cervical, pancreatic, colorectal, endometrial, esophageal, liver cancer, gliomas and seminomas [283-284].

Considering the role of HIWI genes in stem cell self-renewal and its overexpression in a variety of cancers, Liang et al. investigated the effect of HIWI gene silencing in lung cancer, hypothesizing that HIWI knockdown in lung cancer stem cells might exhibit anti-tumor effects. They constructed shRNA eukaryotic expression vector to target HIWI gene *in vitro*, revealing that growth of the lung cancer stem cells was inhibited and the proliferation rate was decreased following HIWI gene silencing [285].

More recently, Liang et al. developed an intravenous delivery system of shRNA plasmids against HIWI, showing a decreased number of lung cancer stem cells and a significant suppression of tumor growth in nude mice [286].

Certainly, the discovery of PIWI proteins associated RNAs has opened a new scenario in gene expression regulation, furthermore, their involvement in genomic stability, gene silencing, DNA repair and several tumorigenic processes, makes this new class of non-coding RNAs particularly interesting, and their potential clinical use, in terms of diagnostic, prognostic and predictive significance should be assessed.

### Long non-coding RNA in lung cancer

In recent years, genome-wide transcriptomic analyses have clarified that the genome of mammals produces many long transcripts, also known as long non-coding RNAs (lnc-RNAs) [287]. LncRNAs are greater than 200 nucleotides long non-protein coding RNAs, mainly transcribed by RNA polymerase II from intergenic regions, promoter regions, or by transcriptional active pseudogenes, [288]. As protein coding genes do, so lncRNAs undergo post transcriptional processing, including 5′capping, alternative splicing, RNA editing, and polyadenylation [288, 5]. Based on their characteristics, lncRNAs are currently divided into five groups which include sense, antisense, bidirectional, intronic, and intergenic [289]. Recent studies have also shown that most lncRNAs displayed tissue-specific expression patterns [290].

When it comes to the various functions performed by lncRNA, the regulation of protein-coding genes expression is certainly the most important, however many others, such as epigenetic regulation, alternative splicing, RNA maturation and transport, protein synthesis, modulation of protein activity, alternation of protein localization, and the fact that they are precursors of small RNAs and tools for silencing miRNAs, have all been well documented (Figure 3) [291-292]. Additionally, lncRNAs are involved in the cell cycle regulation, survival, migration, and metabolism, and their dysregulation appears to contribute to the development and progression of human cancers (Table 4) [293-295].

**Table 4:** Examples of dysregulated long non coding RNAs in lung cancer cancer

Long non-coding RNA	Genomic location	Expression	Clinical implication
MALAT1	11q13.1	Upregulated	Poor prognosis Shorter overall survival Metastasis development
HOTAIR	12q13.13	Upregualated	Poor prognosis Advanced stage Lymph node metastasis Shorter disease free survival
CCAT2	8q24.21	Upregulated	Poor prognosis Lymph node metastasis
BANCR	9	Downregulated	Poor prognosis Advanced stage Shorter overall survival Metastasis development
GAS5/GAS6-AS1	1q25.1/13q34	Downregulated	Poor prognosis Advanced stage Lymph node metastasis Shorter overall survival
ZXF1	10q23.31	Upregulated	Lymph node metastasis Tumor pathological stage Shorter overall survival
Sox2ot	3q26.33	Upregulated	Poor prognosis Metastasis development
CARLo-5	8q24.21	Upregulated	Poor prognosis Metastasis development

H19 was the first lncRNA to be identified, and is expressed exclusively from the maternal allele. Its downregulation has been shown to decrease breast and lung cancer cell clonogenicity and anchorage-independent growth, whereas the loss of imprinting at the H19 locus, and resulting overexpression, has been described in many other cancers, including esophagus, colon, liver and bladder [296]. Interestingly, Lovejoy et al. determined that c-MYC, which is often overexpressed in lung cancer, directly binds to the H19 promoter and highly upregulates the transcription of the maternal H19 allele. These authors also found a strong association between c-MYC and H19 transcript levels, in both primary breast and lung cancer patient material [297].

MALAT1 (metastasis-associated lung adenocarcinoma transcript 1), also known as NEAT2 (nuclear-enriched abundant transcript 2), is the first lcnRNA associated with a strong metastatic potential and poor prognosis in patients affected by non-small cell lung cancer [298]. Currently, it is believed that the full length MALAT1 RNA is processed by RNaseP and RNaseZ to generate the small ncRNA mascRNA, which is subsequently exported to cytoplasm. Conversely, the large MALAT1 RNA is located in the nuclear speckles, where it is involved in alternative splicing [296].

As mentioned previously, MALAT1 high expression was at first identified as a prognosis factor for metastasis and survival in patients with early stage lung adenocarcinomas [298], however, also in squamous cell lung cancer MALAT1 overexpression is associated with a poor prognosis, correlating with an increased cell growth and colony formation of NSCLC cells *in vitro* [300].

Further studies evaluated the role of MALAT1 in the regulation of motility-related genes and metastasis phenotype in lung cancer cells, definitively establishing its role in the processes of cell migration and metastasis, also defining a more malignant neoplastic phenotype [301- 302].

The growing interest in non-invasive diagnostic techniques that may permit an early diagnosis, has led to the evaluation of lncRNAs in blood samples; for instance, HULC was found detectable in blood of patients with hepatocellular carcinoma [303], while PCA3 (prostate cancer gene 3) was found in the urine of prostate cancer patients [304]. Similarly, Weber et al. evaluated the potential role MALAT1 as a blood-based biomarker for NSCLC, finding a sensitivity of 56% and specificity of 96% in distinguishing cancer patients and cancer-free controls. The sensitivity to discriminate squamous cell carcinomas from controls was higher (63%) than the sensitivity to discriminate adenocarcinomas from controls (48%), and no impact of tumor stage, age, gender, and smoking status on MALAT1 levels was observed. Because of its low sensibility and high specificity, MALAT1 detection in blood samples might be used as a complementary biomarker within a panel to improve lung cancer diagnosis [305].

HOTAIR (Hox transcript antisense intergenic RNA) is a 2158 bp long lncRNA located at the HOXC locus on chromosome 12q13.13 [306], principally involved in epigenetic repression of target genes. Once spliced and polyadenilated, this lncRNA binds and recruits PRC2 and LSD1, redirecting the whole complex to HOXD locus on chromosome 2, thus silencing a gene cluster involved in metastasis suppression through H3K27 methylation and H3K4 demethylation [295-296]. Actually, HOTAIR was also identified as a scaffold for the assembly of chromatin-modifying complexes [307].

To date, the negative prognostic influence of HOTAIR increased expression has been described in different types of cancer, such as melanoma, lung, colon, liver, pancreatic, and especially breast cancer [307-308].

As regards lung cancer, Liu et al. found that HOTAIR was highly expressed in NSCLC tissues and NSCLC cell lines. Moreover, they observed that high HOTAIR expression levels were associated with an advanced stage, lymph node metastasis, and poor prognosis, whereas patients with a lower expression of HOTAIR experienced a longer overall survival. Interestingly, this study also demonstrated that RNAi-mediated suppression of HOTAIR decreased the migration and invasion of NSCLC cells *in vitro* and blocked cell metastasis *in vivo*, therefore also suggesting a potential therapeutic role of lncRNA targeted therapies [309]. This data was confirmed and expanded upon by Nakagawa et al., who, by examining the expression of HOTAIR in 77 NSCLCs and 6 brain metastases by quantitative real-time RT-PCR, showed that high levels of expression of HOTAIR were associated with advanced stages of disease, lymph node metastasis and a shorter disease-free interval. It should be emphasized that in brain metastases HOTAIR levels were higher compared to primary tumor tissues [310].

Qui et al. analyzed the expression profile of a novel lncRNA, CCAT2 (colon cancer-associated transcript 2), in lung cancers, noticing that CCAT2 was significantly over-expressed in NSCLC tissues; in particular, they found that CCAT2 overexpression was associated with adenocarcinomas (P=0.033) but not with squamous cell carcinoma. Furthermore, in the same study it was shown that CCAT2 combined with CEA could predict lymph node metastasis, and siRNA silencing of CCTA2 resulted in the inhibition of the proliferation and invasion in NSCLC cell lines [311].

Another emerging lncRNA is the BRAF-activated non-coding RNA (BANCR), a 693-bp lncRNA located on chromosome 9, already found to be involved in melanoma cell migration [312]. Sun et al. evaluated BANCR expression in 113 NSCLC tissues and seven NSCLC cell lines, using quantitative polymerase chain reaction assay. They showed that BANCR was significantly downregulated in NSCLC samples, correlating to a more advanced stage of disease, development of widespread metastases, shorter overall survival, and poor prognosis (independent predictive value for TNM stage, P = 0.038) [313].

Recently, Shi X. et al. determined the expression pattern of the growth arrest-specific transcript 5 (GAS5) in 72 NSCLC specimens by qRT-PCR, assessing its biological role in the development and progression of NSCLC. This study also revealed that GAS5 expression is downregulated in NSCLC tissues compared to adjacent noncancerous tissues (P < 0.05) and is still related to TNM stage (P < 0.05) [171]. Therefore, GAS5 appears to act as a tumor suppressor in NSCLC, by inducing p53 mediated apoptosis and through E2F1 downregulation [314].

Similarly, lncRNA GAS6-AS1 (GAS6 antisense RNA 1) downregulation was found in NSCLC tissues compared with adjacent normal tissues (P < 0.001), also negatively correlating with lymph node metastasis (P = 0.032) and advanced tumor node metastasis stage (P = 0.003). GAS6-AS1 expression was also an independent predictor for overall survival (P = 0.036) [315].

Zhang et al. observed that the expression of long non-coding RNA ZXF1 was significantly upregulated in lung adenocarcinoma tissues compared with adjacent non-cancerous lung tissues (P<0.05) in 62 samples, and correlated with lymph node metastasis (P<0.05), tumor pathological stage (P<0.05), and shorter overall survival [316].

Furthermore, Hou Z et al. found that the expression level of Sox2ot (Sox2 overlapping transcript) was significantly higher in SCCs than that in adenocarcinoma and high Sox2ot expression levels predicted poor survival in lung cancer patients [317].

Lastly, Luo J et al. showed a significant upregulation of CARLo-5 (Cancer-associated region long non-coding RNA) in non small cell lung cancer tissues compared to their adjacent normal tissues, also experiencing a poorer prognosis in those patients with high CARLo-5 expression levels [318].

In summary, all of this data suggests that, in addition to their implication in the pathogenetic mechanisms of lung cancer, lncRNAs may also have a diagnostic and prognostic significance. Although further studies are required to confirm these findings, certainly the world of lncRNA is expected to play an increasingly important role in defining different aspects of this complex disease.

## CONCLUSION AND PERSPECTIVES

The discovery of ncRNA has radically changed and improved our understanding of the molecular mechanisms behind the regulation of gene expression, also greatly enriching our knowledge of the molecular and cellular processes involved in cancer. The amazing versatility of some ncRNA, in particular miRNAs, to serve as diagnostic biomarkers, also with a clear prognostic and predictive value, is leading to the confirmation of ncRNAs as powerful clinical tools. In addition, the possibility of using this new class of RNA either as a therapeutic target and therapeutic means alongside the conventional therapies, justifies the amount of attention that the ncRNAs are receiving.

In this paper we reviewed the functional role of different classes of ncRNA in lung cancer, which represents the leading cause of cancer-related death worldwide. Lung cancer is a complex disease, and currently includes a wide variety of different diseases, each with its own clinical and biological features.

Therefore, it is clear that the study and evaluation of both coding and non-coding transcripts play an important role in defining the pathogenesis of lung cancer. It will enable us to develop a comprehensive clinical approach, also allowing for a further stratification of patients, and thus guarantee the development of targeted therapies based on single patient disease biology. Of course, to achieve these results some technical problems must be solved, like determining which are the best methods of delivering ncRNAs for therapeutic purposes (miRNAs/siRNAs), identifying therapeutic dosage regimes, developing ncRNAs-based diagnostic panels (even across multiple classes of ncRNAs), confirming the prognostic and predictive significance of ncRNAs in large cohort studies and improving non-invasive diagnostic techniques that allow for the early detection of lung cancer.

In conclusion, the study of what has been described as “dark genomic matter” has led to a comprehensive overview of the human genome by shining light on corners of a universe as yet unexplored, but that looks extremely promising for the future applications it could well have to cancer research as well as other diseases.
